# Biomarker and pathogenicity claims for antiphospholipid antibodies in post-treatment Lyme disease syndrome

**DOI:** 10.1128/iai.00436-26

**Published:** 2026-10-20

**Authors:** Armin Alaedini, Gary P. Wormser, Mary K. Crow

**Affiliations:** 1 Department of Medicine, Columbia University Irving Medical Center 21611, New York, New York, USA; 2 Division of Infectious Diseases, Department of Medicine, New York Medical College 8137, Valhalla, New York, USA; 3 Inflammation and Autoimmunity Research Program, Hospital for Special Surgery and Weill Cornell Medicine 25062, New York, New York, USA; Tulane University, New Orleans, Louisiana, USA

**Keywords:** Lyme disease, *Borrelia burgdorferi*, post-treatment Lyme disease syndrome, autoantibody, biomarker, antiphosphatidylserine antibodies, antiphospholipid antibodies, autoimmunity

## LETTER

We read with interest the recent article by Shrestha et al. examining antiphospholipid antibodies in acute Lyme disease and post-treatment Lyme disease syndrome (PTLDS) (1). The study provides valuable longitudinal data on antiphospholipid antibody responses following *Borrelia burgdorferi* infection. We believe, however, that several conclusions in the article regarding biomarker utility, disease specificity, and pathogenicity are not fully supported by the analyses presented.

The abstract concludes that “a subset of patients with chronic symptoms is identifiable by persistent elevation in antiphosphatidylserine” and suggests that these antibodies “could serve as a biomarker for chronic disease.” A biomarker for PTLDS, however, should distinguish patients with persistent symptoms following treated Lyme disease from those who returned to health after treatment. Previous studies examining antibody reactivity to self-antigens in PTLDS have incorporated post-Lyme recovered comparator groups (2, 3). Although the present study included an appropriate longitudinal return-to-health (RTH) cohort, the antibody trajectories in the PTLDS and RTH groups were broadly similar throughout the first year after infection. The authors specifically state that, at 12 months, the differences between the groups were not significant for either antiphosphatidic acid or antiphosphatidylserine IgG. Consequently, the presented analyses do not establish that antiphosphatidylserine antibodies distinguish PTLDS from recovery following Lyme disease or support the conclusion that they could serve as biomarkers of chronic disease. Likewise, although the abstract states that “a subset of patients with chronic symptoms is identifiable,” the study does not define or test such a subset or establish that it differs from recovered Lyme participants.

Similarly, the presented analyses do not demonstrate that antiphosphatidylserine antibodies distinguish PTLDS from clinically similar conditions or autoimmune diseases. The abstract states that antiphosphatidylserine antibodies identified in PTLDS “were not found in a panel of sera from patients with look-alike autoimmune disorders.” However, the reported statistical analyses compare each disease group with healthy controls rather than directly with PTLDS. A significant difference between PTLDS and controls, together with a nonsignificant difference between another disease group and controls, does not establish a significant difference between PTLDS and that disease group. The limited sizes of the comparator cohorts further constrain conclusions regarding disease specificity. Furthermore, IgG antibodies directed against phosphatidylserine have previously been described in systemic lupus erythematosus and other autoimmune conditions (4–6). Therefore, the presented findings do not support the conclusion that antiphosphatidylserine antibodies are specific for PTLDS.

Finally, the abstract states that the study was undertaken “to assess the potential contribution of antilipid antibodies to Lyme disease pathology” and concludes that persistent antiphosphatidylserine antibodies “may drive autoimmune-like symptoms.” The experiments, however, measure antibody levels but do not test whether these antibodies contribute to disease. No associations between antibody levels and symptom severity, no functional characterization of the antibodies, and no mechanistic evidence supporting a pathogenic role are presented. Accordingly, the study examines the association between antibody levels and selected study groups but does not assess whether these antibodies contribute to disease pathogenesis.

These considerations do not detract from the reported observations themselves. Rather, they emphasize the importance of ensuring that conclusions regarding biomarker utility, disease specificity, and mechanistic inference remain proportionate to the evidence presented, particularly in the title and abstract, where they are most likely to shape interpretation by readers. Maintaining this evidentiary standard will be essential for guiding future studies toward biomarkers and mechanisms that are biologically informative and clinically meaningful.

## EDITOR'S NOTE

**Ed. Note**: The authors of the original article did not respond at the time of publication.
